# Transit peptide elements mediate selective protein targeting to two different types of chloroplasts in the single-cell C4 species *Bienertia sinuspersici*

**DOI:** 10.1038/srep41187

**Published:** 2017-01-23

**Authors:** Diana Wimmer, Philipp Bohnhorst, Vinay Shekhar, Inhwan Hwang, Sascha Offermann

**Affiliations:** 1 Institute for Botany, Leibniz University Hannover, Herrenhaeuser Strasse 2, Hannover 30419, Germany; 2 Faculty of Biology, Department Biology I – Botany, Ludwig-Maximilians-University Muenchen, Grosshaderner Strasse 2-4, 82152 Planegg-Martinsried, Germany; 3 Division of Integrative Biosciences and Biotechnology, Pohang University of Science and Technology, Pohang 790–784, Korea

## Abstract

*Bienertia sinuspersici* is a terrestrial plant that performs C4 photosynthesis within individual cells through operating a carbon concentrating mechanism between different subcellular domains including two types of chloroplasts. It is currently unknown how differentiation of two highly specialized chloroplasts within the same cell occurs as no similar cases have been reported. Here we show that this differentiation in photosynthetic cells of *B. sinuspersici* is enabled by a transit peptide (TP) mediated selective protein targeting mechanism. Mutations in the TPs cause loss of selectivity but not general loss of chloroplast import, indicating the mechanism operates by specifically blocking protein accumulation in one chloroplast type. Hybrid studies indicate that this selectivity is transferable to transit peptides of plants which perform C4 by cooperative function of chloroplasts between two photosynthetic cells. Codon swap experiments as well as introducing an artificial bait mRNA show that RNA affects are not crucial for the sorting process. In summary, our analysis shows how the mechanism of subcellular targeting to form two types of chloroplast within the same cell can be achieved. This information is not only crucial for understanding single-cell C4 photosynthesis; it provides new insights in control of subcellular protein targeting in cell biology.

Loss of carbon through photorespiration is common in C3 plants especially in warm or dry environments which results in significant decrease in growth and harvestable yields^1^. Photorespiration is initiated when the enzyme responsible for assimilation of carbon dioxide, ribulose 1,5-bisphosphate carboxylase/oxygenase (Rubisco) reacts with oxygen instead of CO_2_. Rubisco’s oxygenation versus its carboxylation activity increases when the intracellular CO_2_ to O_2_ ratio decreases as a result of stomatal closure in response to drought or heat stress^2^, and because specificity of Rubisco for CO_2_ declines with increasing temperature^3^. In order to overcome problems associated with photorespiration, in some plant families, CO_2_ concentrating mechanisms evolved, including different forms of C4 photosynthesis. C4 photosynthesis can outperform C3 photosynthesis especially under photorespiratory conditions^4^ and this created considerable interest in implementing a C4 cycle into C3 crops such as rice to improve yields and stress tolerance^5^,^6^.

The C4 cycle functions to capture atmospheric CO_2_ in one domain (by PEP carboxylase), and concentrate CO_2_ in another domain (by decarboxylation of C4 acids) where the CO_2_ is then assimilated by Rubisco in the Calvin-Benson-Bassham (CBB) cycle. The vast majority of all known C4 plants use a CO_2_ concentrating mechanism based on a dual cell arrangement (Kranz anatomy) with spatial separation of primary and secondary CO_2_ fixation reactions in two different cell types (mesophyll cells (MC) and bundle sheath cells (BSC)). However, a remarkable exception was discovered in the family Amaranthaceae. Here, a total of four species are known to perform a complete C4 cycle within individual photosynthetic cells (single-cell C4, SCC4)^7^,^8^,^9^,^10^. This is enabled by a unique subcellular compartmentalization which to date has not been observed for other plants. Two structural variants of SCC4 exist: In *Suaeda aralocaspica*, two morphologically, biochemically and physiologically different types of chloroplast are located at opposing poles of the photosynthetic cells^9^,^11^, whereas in the three other species (all members of the genus *Bienertia*), one chloroplast type (central (C) chloroplasts) is located in an internal compartment at the center of the cell and the other chloroplast type is located in the periphery close to the plasma membrane (peripheral (P) chloroplasts)^7^,^8^,^10^,^12^. Most information on single-cell C4 photosynthesis comes from the model species *Bienertia sinuspersici* (hereafter referred to as Bienertia). In Bienertia, it has been shown that the two chloroplast types are separated by a single large vacuole^13^, which presumably serves as a diffusion barrier between the two reaction compartments. The peripheral and the central compartments are connected by numerous cytoplasmic channels for metabolite exchange^8^,^14^. The unique subcellular compartmentalization and the specialized biochemistry of the two chloroplast types observed in SCC4 species develop gradually during ontogenesis. In Bienertia, very young cells located at the base of young leaves show only a single chloroplast type that operates in a “default” C3 photosynthetic mode^14^,^15^. Towards maturation, the two chloroplast types and the peripheral and central domain develop through exposure to yet unknown signals. At the tip of young leaves, the two domains have completely separated, together with biochemical specialization and a fully functional C4 cycle within these cells^14^.

Numerous studies have demonstrated that the two chloroplast types accumulate different sets of nuclear encoded proteins according to their specific functions in the C4 and C3 cycle respectively^8^,^9^,^15^,^16^,^17^,^18^. For example, nuclear encoded pyruvate, Pi-dikinase (PPDK), the key enzyme needed for generation of the primary CO_2_ acceptor phospho*enol*pyruvate (PEP) is found specifically in the P-chloroplasts only. In contrast, P-chloroplasts lack Rubisco and a functional CBB cycle, which only operates in the C-chloroplasts. All currently known SCC4 species belong to the NAD-ME C4 subtype where decarboxylation of C4 acid occurs exclusively in mitochondria which in the case of Bienertia are restricted to the central compartment^15^,^19^. CO_2_ released from mitochondrial decarboxylation can then be re-fixed by the adjacent C-chloroplasts. In summary, protein localization studies, gas exchange properties and physiological experiments indicated that the P- and C-chloroplasts of SCC4 species basically resemble the biochemistry and function of MC and BSC chloroplasts of Kranz C4 species^16^,^17^,^20^,^21^.

In Kranz C4 species, it has been shown that many nuclear encoded proteins, either directly or indirectly related to their specific function in C4 photosynthesis, accumulate either in MC or BSC chloroplasts^22^. Large-scale expression data indicate that chloroplast type specific protein accumulation patterns can be explained mostly by cell type specific expression differences between MC and BSC^23^. Since the two chloroplasts of Bienertia develop and specialize within individual cells, tissue specific transcriptional control cannot account for the biochemical specialization of the two chloroplast types. Instead, a posttranscriptional mechanism must exist that ensures the correct targeting of proteins either to the P- or C-chloroplasts. It has been speculated that such a mechanism could work either by selective mRNA targeting, selective protein import into the chloroplasts or selective degradation within the two chloroplast types^24^,^25^ but as of now, there is no experimental evidence on the nature of this mechanism.

Here we provide a detailed analysis of the targeting characteristics of recombinant fusion proteins in the SCC4 species *Bienertia sinuspersici*. We show that selective targeting to the peripheral chloroplasts is mediated by specific sequence elements within the transit peptide which are distinct from elements required for general chloroplast import. We provide the first experimental evidence on how subcellular targeting to specialized chloroplasts works and on how SCC4 species can use this to perform C4 photosynthesis within individual cells.

## Results

### Chloroplast type specific targeting can be replicated *in vivo* in young but not in mature protoplasts

**Figure 1:**
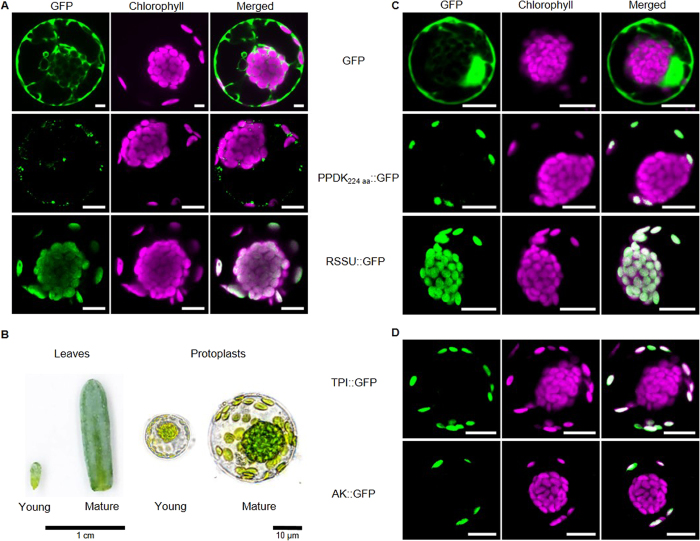
Subcellular localization of chloroplast targeted proteins in different developmental stages of *Bienertia sinuspersici*. (**A**,**C**,**D**) Confocal images of various transiently expressed GFP-fusion proteins in *B. sinuspersici* chlorenchyma protoplasts. All fluorescence images are shown in the GFP channel (green - excitation 488 nm/emission 509/525 nm) and chlorophyll autofluorescence (red - excitation 408 nm/emission 620/700 nm). Additionally, the merged channels are shown. All images are representative from n ≥ 5 independent experiments. All scale bars = 10 μm. (**A**) Transient expression of GFP, PPDK_224_-GFP and full length RSSU-GFP in mature protoplasts. (**B**) Size comparison between young (Y) and mature (M) leaves and protoplasts. Scale bar leaves = 1 cm; Scale bar protoplasts = 10 μm. (**C** + **D**) Transient expression of GFP, PPDK_224_-GFP, RSSU-GFP, TPI-GFP and AK-GFP in young protoplasts. GFP – green fluorescent protein; PPDK – Pyruvate, Pi dikinase; RSSU – Rubisco small subunit; TPI – triosephosphate isomerase; AK – adenylate kinase.

Rubisco small subunit (RSSU) and PPDK are key enzymes operating in the CBB- and the C4 cycle, respectively. Their preferential accumulation in the P- and C-chloroplasts has been demonstrated previously by *in situ* localization studies^8^,^15^ as well as western blot analysis^17^ and proteomics^16^. Thus, we used these two markers as a starting point to analyze the mechanism behind selective protein accumulation in Bienertia. A full length precursor of RSSU (RSSU::GFP) and a precursor consisting of the first 224 out of 951 amino acids (AA) of PPDK (PPDK_224_::GFP) were fused to GFP and tested in protoplasts isolated from mature Bienertia chlorenchyma cells. Neither construct showed chloroplast type specific accumulation. Instead, GFP appeared in a speckled pattern, presumably localized in the cytoplasm in the case of PPDK_224_::GFP or accumulated in both chloroplast types in the case of full length RSSU::GFP (Fig. 1A). To test whether the utilization of a non-full length construct in the case of PPDK interfered with general chloroplast import, the same construct was used to transfect *Arabidopsis thaliana* protoplasts (Fig. S1). Here, PPDK_224_::GFP was imported correctly into chloroplasts indicating that the observed mistargeting in mature Bienertia protoplasts is a Bienertia specific effect.

We also tested the influence of different light intensities, temperatures and buffer conditions during the transient expression procedure, as well as inclusion of the 5′UTR regions and testing of different RSSU isoforms. None of it resulted in a noticeable difference in the protein accumulation pattern compared to those observed in Fig. 1A. However, when the same constructs were tested side-by-side in protoplasts prepared from an earlier developmental stage (young chlorenchyma cells Fig. 1B) we observed strikingly different results for PPDK_224_::GFP, since it was now localized predominantly in the P-chloroplasts (Fig. 1C). A similar localization pattern was observed for adenylate kinase (AK) which together with PPDK is involved in regeneration of PEP (Fig. 1D). We also tested localization of chloroplastic triose-phosphate isomerase (TPI). TPI is typically associated with the CBB-cycle and as such, it might not be expected in P-chloroplasts. However, C4 plants are able to export part of the 3-phosphoglycerate produced by the CBB-cycle to the mesophyll chloroplasts (in case of Kranz C4) or to the P-chloroplasts (in case of Bienertia) where it is reduced to glyceraldehyde 3-phosphate (G3P). After conversion by TPI to dihydroxyacetone phosphate (DHAP), triose-phosphates can then shuttle back to the bundle sheath (in case of Kranz C4) or the C-chloroplasts (in case of Bienertia) as part of the triose-phosphate shuttle (also called “open Calvin cycle”). In Bienertia, the most abundant isoform of TPI was shown to be strongly enriched in the P-chloroplasts^16^ and its GFP fusion showed a localization pattern similar to that of PPDK and AK (Fig. 1D). In contrast, full length RSSU::GFP showed no indication for C-specific accumulation even in young protoplasts (Fig. 1C). Accordingly, correct localization of at least the P-targeted proteins in *in vivo* localization studies is dependent on the developmental stage or age of the utilized protoplasts, since correct targeting was observed in young but not mature protoplasts.

### The transit peptide contains all information required for P-specificity

**Figure 2:**
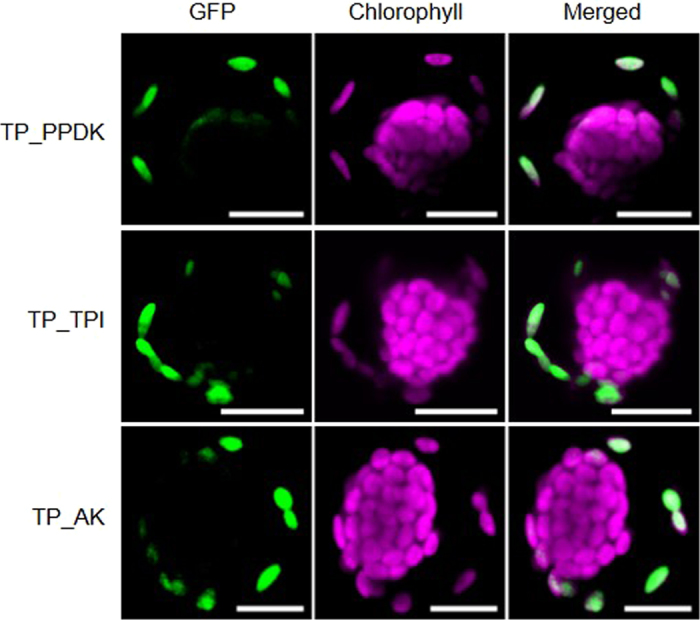
Subcellular localization of transit peptide GFP-fusions in young *Bienertia sinuspersici* protoplasts. Representative confocal images for all tested GFP-fusion constructs (n > 5) of GFP-fusion proteins of the P-specific proteins PPDK, TPI and AK in chlorenchyma protoplasts (TP_PPDK, TP_TPI, TP_AK). Transit peptides length was predicted by ChloroP^55^ (Table S5). Protoplasts were analyzed as described in Fig. 1. Scale bars = 10 μm. GFP - green fluorescent protein; PPDK – Pyruvate, Pi dikinase; TPI – triosephosphate isomerase; AK – adenylate kinase.

To further narrow down the elements necessary for P-specific protein accumulation, we tested if transit peptide (TP)::GFP-fusions of PPDK, AK and TPI (referred to below as TP_PPDK, TP_AK and TP_TPI) are sufficient for P-specific targeting (Fig. 2). In all cases tested, the efficiency in delivering GFP specifically to the P-chloroplasts was similar to the previously tested full length constructs (see Fig. 1C and D). This indicates that the TP already contains all the necessary information.

### Discrete elements in the TP control either general import or P-specific import

**Figure 3:**
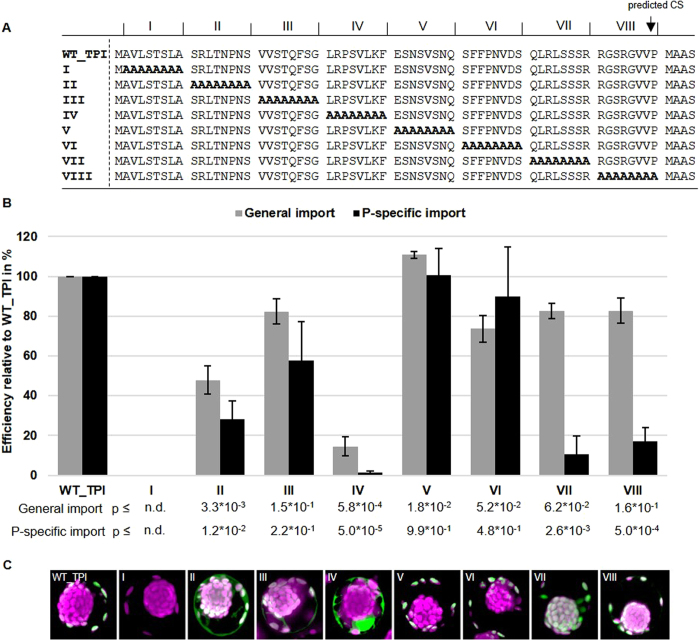
Distinct sequence elements in the transit peptide of TPI correlate with either general or P-specific chloroplasts import. (**A**) Sequences of WT_TPI and corresponding alanine substitution mutants. CS – cleavage site; TPI – triosephosphate isomerase. (**B**) Quantification of GFP signals in transfected protoplasts (WT_TPI; I – VIII mutant GFP-fusion constructs). Transfected protoplasts were counted and the expression pattern of GFP was categorized in general or P-specific import into the chloroplasts and shown as import efficiency relative to WT_TPI in percent. Quantification was performed as a blind study to reduce subjective bias as explained in Material and Methods. The means of four independent biological replicates are shown relative to WT_TPI. Error bars indicate standard error of the mean. Numbers indicate p-values from Student’s t-test. (**C**) Representative confocal images for all tested GFP-fusion constructs in young protoplasts (n > 5).

Next, we analyzed whether discrete elements within the TPs can be identified that control P-specificity. Therefore, we constructed mutant TPI TP sequences where clusters of eight amino acids were sequentially substituted with alanines (Fig. 3A) under the assumption that this substitution would interfere with the inherent function of the underlying AA sequence. Mutations in cluster I generally resulted in no observable GFP expression, probably through interference of the induced mutations with translation. Compared to the wild type transit peptide TPI sequence (WT_TPI), we observed a strong reduction in P-specificity when the clusters II, IV, VII and VIII were mutated (Fig. 3B for a quantitative analysis and Fig. 3C for a representation of the observed phenotype). However, we also observed that in clusters II and IV the general import efficiency (that is import in either P- or C-chloroplasts) was severely reduced (by more than 50 and 80 percent in clusters II and IV, respectively) as the majority of GFP signal was found in the cytoplasm (Fig. 3C panels II and IV). Accordingly, the two elements located in clusters II and IV are required for general import and the two elements located in clusters VII and VIII are required for P-specificity.

### The primary sequence for the P-specific elements is not conserved between different P-targeted transit peptides

**Figure 4:**
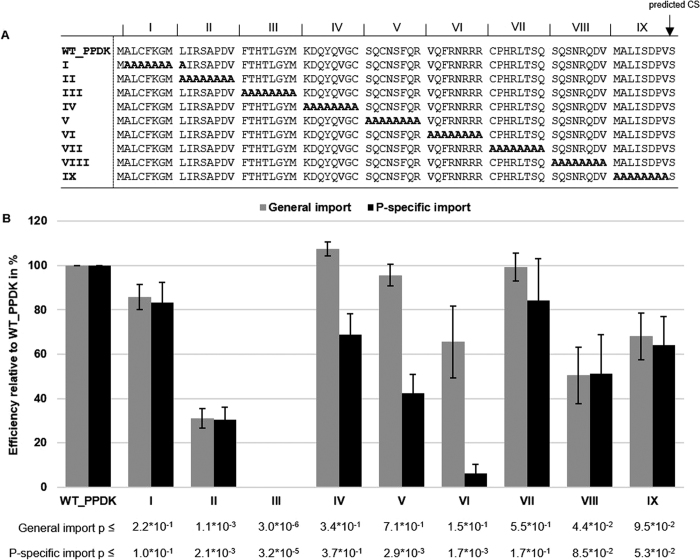
Distinct sequence elements in the transit peptide of PPDK correlate with either general or P-specific chloroplasts import. (**A**) Sequences of WT_PPDK and corresponding alanine substitution mutants. CS – cleavage site; PPDK – Pyruvate, Pi dikinase. (**B**) Quantification of GFP signals in transfected protoplasts (WT_PPDK; I – IX mutant GFP-fusion constructs) by fluorescence microscopy. Expression patterns were categorized and analyzed as described in Fig. 3 (n = 3). p-values are indicated in the figure.

To test, whether transit peptides of other P-targeted proteins contain similar elements as observed for the WT_TPI we repeated the analysis with the transit peptide of PPDK (Fig. 4). Similarly, we observed elements that when mutated reduced the overall import efficiency (for example clusters II and III). Mutations in two other clusters (V and VI) reduced P-specific import significantly but did not affect general import efficiency much, indicating a generally similar setup for the two TPs analyzed. However, comparing the AA sequences of the P-specific clusters VII and VIII from the WT_TPI (Fig. 3) with the P-specific clusters V and VI from this experiment revealed no obvious conservation of the primary AA sequence.

### Targeting specificity is insensitive to changes in the mRNA sequence coding for the transit peptide

**Figure 5:**
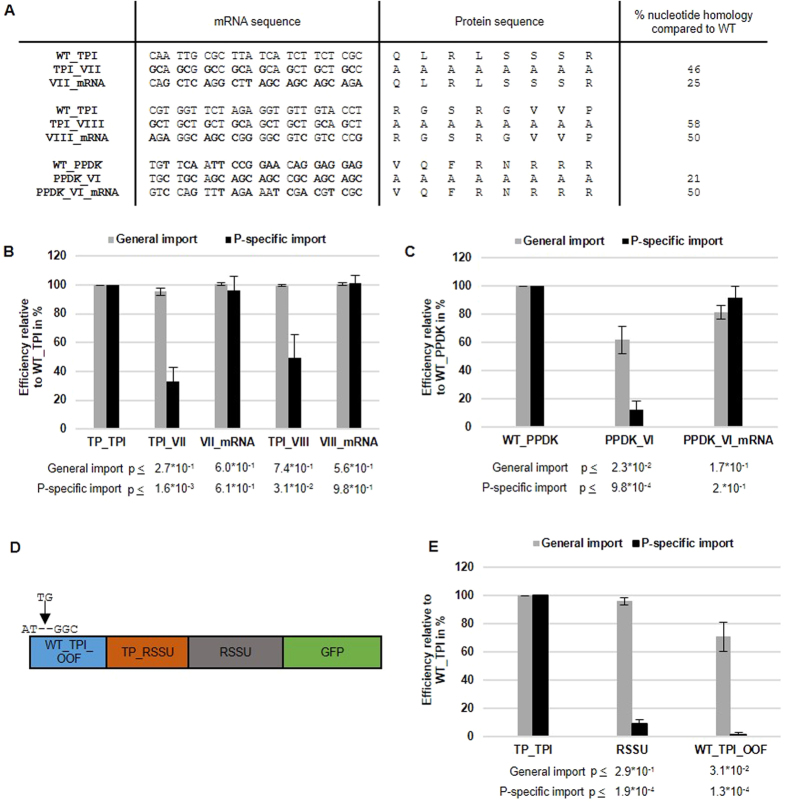
Mutations affecting the mRNA sequence but not the amino acid sequence of the transit peptides of TPI or PPDK do not affect P-specificity. (**A**) Partial mRNA sequences and corresponding amino acid sequences from wild type TPs (WT_TPI, WT_PPDK), their alanine substitutions (TPI_VII, TPI_VIII, PPDK_VI) and the wobbled/codon switched mRNA (VII_mRNA, VIII_mRNA, VI_mRNA). Numerals indicate homology compared to WT in percent. (**B**,**C**,**E**) Quantification of GFP signals in transfected protoplasts by fluorescence microscopy. Expression pattern of GFP was categorized in general or P-specific import. Protoplasts were analyzed and statistics were performed as described in Fig. 3. p-values are indicated in the figure. Efficiency of P-specific and general import of TPI (n = 4) (**B**) or PPDK (n = 3) (**C**) in comparison to the alanine substitution mutants and mRNA mutations. Error bars indicate standard error of the mean. (**D**) Chimeric construct of the nucleotide sequence of the TP of TPI fused in front of the coding region of full length RSSU-GFP. The nucleotide sequence of the TPI transit peptide contains a two nucleotide insertion at the start codon to produce an out of frame (OOF) shift resulting in a non-translatable mRNA sequence for the TPI TP followed by a translatable mRNA sequence for the full length RSSU. (**E**) Efficiency of P-specific and general import of the chimeric construct (n = 3).

Since the alanine substitution experiments did not only affect the amino acid sequence but also the nucleotide sequence of the underlying mRNA coding for the TPs (through substituting all codons to codons for alanine), we cannot formally exclude that the observed effects are mRNA effects rather than TP effects. We therefore designed two experiments to distinguish between these two possibilities. Firstly, we created mutant sequences that contained as many nucleotide changes as possible within the mutated clusters (through wobbling of the third base and usage of alternative codons whenever possible) without affecting the amino acid sequence. Compared to the WT sequences, these mutations showed only between 25% to 50% sequence homology in the clusters VII and VIII for the WT_TPI and the cluster VI of the WT_PPDK, respectively (Fig. 5A). Compared to the alanine substitutions, these mRNA mutations had no effect on the P-targeting specificity for either the WT_TPI or the WT_PPDK sequence (Fig. 5B and C).

Additionally, we created a chimeric construct, consisting of an out-of-frame (OOF) nucleotide sequence for the TP of TPI fused to the (in-frame) full length RSSU including the TP (Fig. 5D). The frame-shifted leader from the P-specific TPI is almost identical (except for a 2 nucleotide insertion as shown in Fig. 5D) to the mRNA nucleotide sequence of the wild-type TPI transit peptide. This construct therefore comprises nearly the identical mRNA nucleotide sequence as the P-specific WT_TPI followed by a non-P-specific but functional full length RSSU (WT_TPI_OOF). Comparison with RSSU shows that the mRNA frame-shifted nucleotide sequence of the WT_TPI is not able to restore the P-specificity (Fig. 5E). In contrast, when the TP of TPI is fused in-frame in front of the TP of RSSU (Fig. S2A) or when the TP of RSSU is placed in-frame in front of the TP of TPI (Fig. S2B), P-specificity is restored.

In conclusion, both the wobble/codon swap experiment as well as the out-of-frame fusions did not indicate an involvement of mRNA effects in the specific targeting of the TPs to the P-chloroplasts. Instead, the amino acid sequence is the determining factor.

### Detailed analysis of the P-specific elements

**Figure 6:**
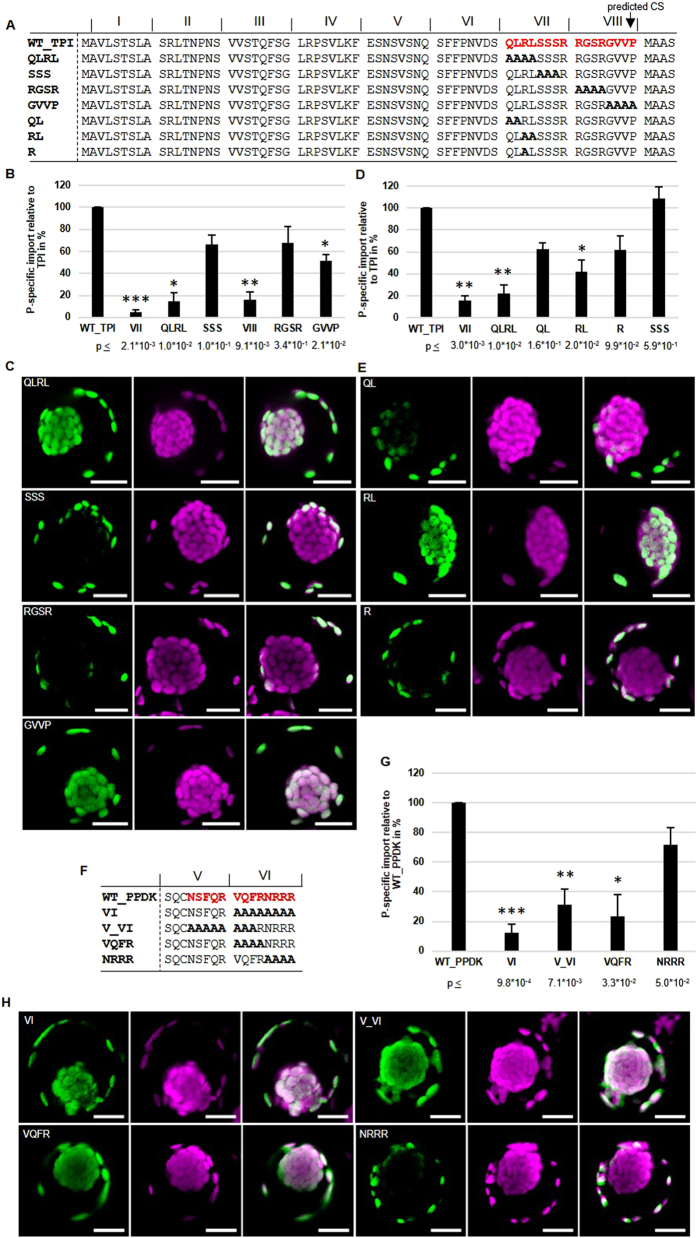
Four amino acids in the transit peptides of TPI and PPDK are necessary for P-specificity. (**A**–**E**) Sequences, quantification and representative images for TPI. (**F**–**H**) Sequences, quantification and representative images for PPDK. (**A**,**F**) Sequences of the wild type TPI and PPDK transit peptide sequences and the corresponding alanine substitution mutants. (**B**,**D**,**G**) Quantification of GFP signals in transfected protoplasts by fluorescence microscopy. Protoplasts were analyzed (n = 3) and statistics were performed as described in Fig. 3. Asterisks indicate significant differences from the corresponding control experiment (WT_TPI or WT_PPDK) by Student’s t-test (*p < 0.05; **p < 0.01; ***p < 0.001). (**C**,**E**,**H**) Representative confocal images of observed phenotypes. For representative images of the phenotypes of WT_TPI and WT_PPDK see Fig. 2. For representative images of the phenotypes of motifs VII and VIII of TPI see Fig. 3).

To determine the minimum sequence requirements for P-specificity, contribution of individual amino acids in clusters VII and VIII of WT_TPI and cluster V and VI of WT_PPDK were analyzed in detail (Fig. 6).

The AA sequences “QLRL” and “SSS” in Cluster VII as well as “RGSR” and “GVVP” in the clusters VIII of the WT_TPI were separately substituted with alanines (Fig. 6B for quantitative data and Fig. 6C for representative phenotypes). P-specificity of the triple S substitution was very similar to the wild type TP. In contrast, substitution of QLRL (Fig. 6C) almost completely abolished P-specificity. For Cluster VIII only the GVVP substitution showed a slight reduction in P-specificity but the effect was much smaller compared to the substitution of the whole cluster VIII. Breaking the QLRL motif further down showed only a moderate and somewhat additive effect in reduction of P-specificity for the QL, RL and R substitutions (Fig. 6D and E). In summary, for the TP of TPI a core element in cluster VII (QLRL) and the whole cluster VIII seem to be essential for P-specificity.

A similar analysis was performed on clusters V and VI on the TP of WT_PPDK (Fig. 6F–H). Because alanine substitution of cluster V also showed a slight reduction in P-specificity (see Fig. 4), a combined substitution with the amino acids “NSFQRVQF” spanning the clusters V and VI was performed. Also, the amino acids “VQFR” and “NRRR” of cluster VI were separately tested. Substitutions of the clusters VI, V_VI and of the “VQFR” sequence showed a significant reduction in P-specificity whereas substitution of the NRRR sequence showed the same P-specificity as the transit peptide of wild type PPDK.

Therefore, both the TP of TPI as well as the TP of PPDK carry short sequences of four amino acids (QLRL and VQFR) in their transit peptide which are essential for P-specificity.

### The identified elements are necessary but not sufficient for P-specificity

**Figure 7:**
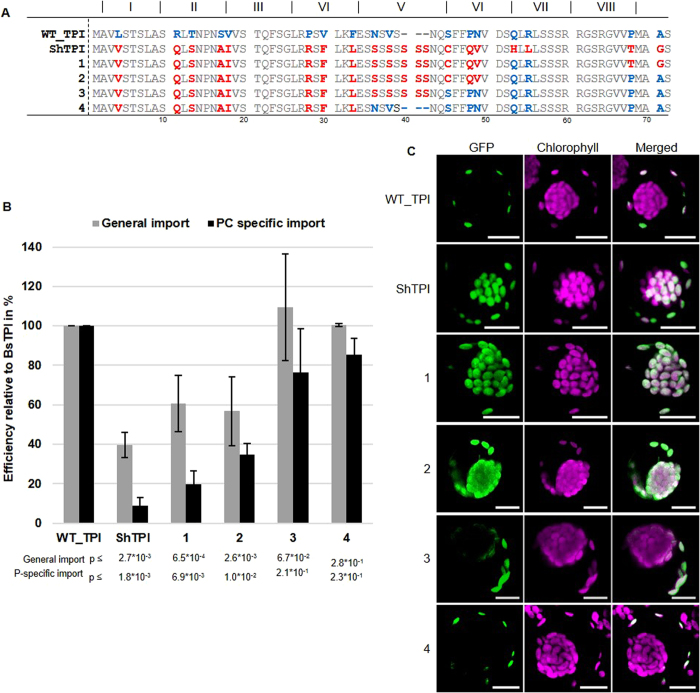
P-specificity from the SCC4 Bienertia TPI transit peptide can be transferred to the TP of the C3 species *Suaeda heterophylla*. (**A**) Sequences of the chimeric TPI TP fusions between Bienertia and *Suaeda heterophylla*. Blue and red letters indicate sequence differences between Bienertia and *S. heterophylla*, respectively. 1–4 indicate different chimeric constructs with varying amounts of sequence information from Bienertia included in the sequence of *S. heterophylla*. (**B**) Quantification of GFP signals in transfected protoplasts by fluorescence microscopy. Expression patterns were categorized and analyzed as described in Fig. 3. p-values are shown in the figure (n = 3). (**C**) Representative confocal images for the GFP-fusion constructs.

The previous experiments demonstrated that the identified elements (QLRL of motif VII and the whole motif VIII) in the TP of TPI are necessary for P-specificity. We further tested if these elements are also sufficient to transfer P-specificity to an unrelated (non P-specific) TP from another species. Therefore, hybrid TPs with the N-terminus of the WT_TPI of the closely related C3 species *Suaeda heterophylla* (from here on Suaeda) and the C-terminus of varying length from the Bienertia WT_TPI (termed BsTPI) were constructed (Fig. 7A). Although the AA sequences of the wild type TPI transit peptides of Suaeda and Bienertia are 80.6% identical, the transit peptide of Suaeda TPI (from here on ShTPI) does not target GFP specifically to the P-chloroplasts when heterologously expressed in Bienertia protoplasts (Fig. 7B and C). Restoring the “QLRL” AA sequence at position 53 and 55 (construct 1) and the AA “P” at position 68 (predicted cleavage site) is not sufficient for restoring P-specificity (construct 2) although this reverts the motifs VII and VIII back to the state as found in WT_TPI from Bienertia. Only when three more AAs at the positions 45, 48 and 49 are converted to the same sequence as in the Bienertia WT_TPI transit peptide P-specificity is fully restored (construct 3). The remaining differences in the N-terminus of the TP are however not important (construct 4), indicating that the P-specific region is clearly confined towards the C-terminus of the TP but specificity seems also to require several additional AAs which are located at some distance from each other.

## Discussion

The C4 carbon concentrating mechanism in Bienertia requires elaborate subcellular compartmentation and accordingly, a protein sorting mechanism for nuclear encoded proteins to the two different chloroplast types. No comparable system is known from either plants or animals for chloroplasts or mitochondria. Therefore, no information on the mechanistic basis of the underlying sorting mechanism was available at the beginning of this study.

For all P-specific proteins tested, we identified the TP as the determining factor for correct localization. In C3 species, TPs have not only been shown to be necessary for correct chloroplast import, but are also able to discriminate between various plastid types in different tissues, e.g. chloroplasts and leucoplasts. This is achieved by a differential TOC/TIC composition as well as different binding properties of TPs^26^,^27^. Furthermore, different substrate-specific import pathways have been identified in chloroplasts, with some precursors using the TOC159 complex whereas others seem to prefer complexes with TOC132^28^,^29^,^30^. These differences in the import pathways are mediated by TPs which prefer different TOC-complexes^31^,^32^. In Bienertia, subcellular localization of the main TOC receptors TOC159/132 and TOC34 has been tested previously by *in vivo* localization studies. However, no preferential accumulation of the fusion constructs in either P- or C-chloroplasts was observed^33^. Therefore, there is currently no evidence for the involvement of these major TOC complexes in the differential sorting process.

**Figure 8:**
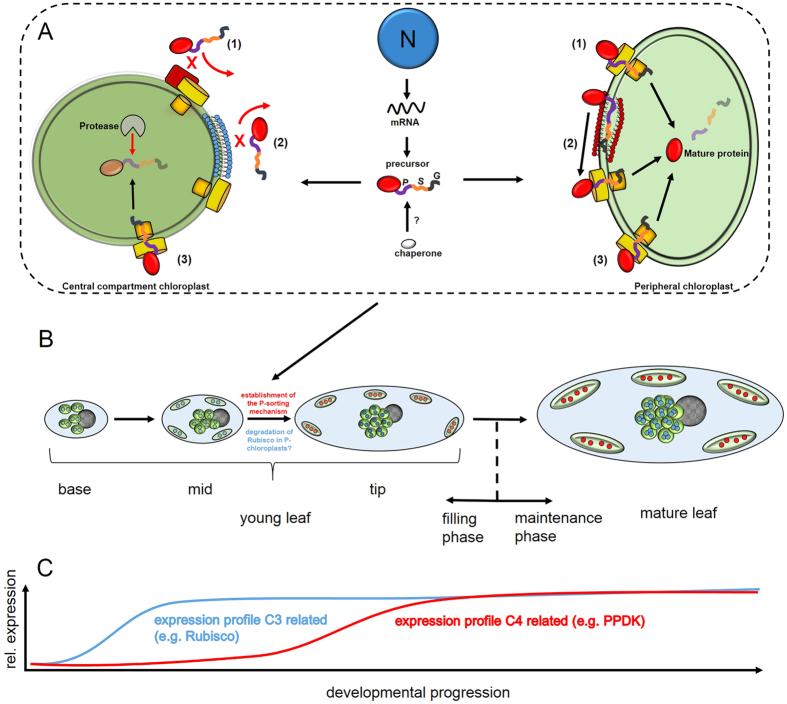
Summary and model for differential accumulation of nuclear encoded proteins within the two different chloroplast types of *Bienertia sinuspersici*. (**A**) Potential mechanisms for P-specific accumulation of nuclear encoded, plastid targeted proteins. Nuclear (N) encoded mRNAs for P-chloroplast specific proteins are translated without location preference. The TP precursors carry elements required for general chloroplast import (G) at the N-terminus and P-specific accumulation (P) at the C-terminus separated by spacers (S) and potentially can also interact with chaperones. Different scenarios could explain the TP mediated, P-specific accumulation: P-specific proteins could be blocked from import into the C-chloroplast via so far unknown components of the TOC-TIC complex (1); The P-element could block interaction with the C-chloroplast envelope (2); The P-element could refer degradation of P-specific proteins within the C-chloroplasts (3). (**B**) Developmental progression of Bienertia chlorenchyma cells. Young cells from the base of young leaves have only a single-chloroplast type that contains Rubisco (blue dots). In the midsections, chloroplasts have started positioning within the peripheral and the central compartment, but all still contain Rubisco along with raising levels of PPDK (modeled after^14^). Towards maturation, the P-specific sorting mechanism is initiated and Rubisco is removed (either by degradation or by preventing re-import) from the P-chloroplasts. In the tip of young leaves, chloroplast positioning and biochemical specialization is completed. (**C**) Simplified accumulation profiles of C3 and C4 related proteins during development (compiled from^8^,^15^,^16^,^18^,^43^. X-axis represents the same developmental stages (base, mid, tip and mature) as in (**B**).

To characterize the specific import pathway in Bienertia, we used extensive alanine scanning, a method that has previously been utilized successfully to identify specific motifs in the TPs of Arabidopsis^34^,^35^,^36^,^37^. Interestingly, our mutation experiments indicate that the identified elements on the TPs of P-specific precursors prevent import into the CC rather than facilitating the specific import into the P-chloroplasts (Fig. 8A). This can be concluded, since deactivation of these elements leads to import into both chloroplast types and not to a complete loss of import which would be expected if the elements are required for specific uptake into the P-chloroplasts. This mechanism might seem surprising, but it makes sense in the greater context: The general proteome of the two chloroplast types is similar in Bienertia^16^ and accordingly, the vast majority of nuclear encoded plastid targeted proteins has to be imported into both chloroplast types. Therefore, information for both P- and C-targeting would be required for all those proteins to ensure equal distribution. Hence, it seems more economical to utilize the general chloroplast import pathway (also indicated by the fact that all tested Bienertia constructs were imported correctly when expressed in Arabidopsis protoplasts) and then prevent the few P-specific proteins from being imported into the C-chloroplasts. Accordingly, certain components that detect and subsequently block the passage of P-specific precursors could be connected to the TOC-TIC machinery of the C-chloroplasts (option 1 in Fig. 8A). The same might also be true for blocking C-specific proteins from import into P-chloroplasts although the latter case remains to be shown.

Historically, TPs have been regarded as being rather unstructured which was also connected to the high variability between different TPs as hardly any common motifs have been identified^38^. However, more recent studies show that TPs are more structured than previously expected. The bimodal model postulates that TPs contain different recognition sites for stromal chaperones and TOC proteins which are coupled by a spacer element^39^. The multi-selection and multi-order (M&M) model extends the bimodal model with the hypothesis that the order of specific motifs can vary among TPs^40^. Comparable to these models, the N-terminus of the TP of P-specific precursors in Bienertia is responsible for general import into chloroplasts, followed by a spacer element (where the alanine substitutions had no effect on either the general or the P-specific import). The C-terminus is then responsible for P-selectivity. It is important to note that the P-specific element at the C-terminus overrides the general import element at the N-terminus. This probably indicates that the P-element was evolutionary acquired later “on top” of the already existing general import mechanism.

Although the identified QLRL and VQFR motifs are necessary for P-specificity, our experiments with Bienertia-*Suaeda heterophylla* hybrids indicate that they are not sufficient, since P-specificity was achieved only when the last 25 aa from the beginning of the C-terminus were restored (Fig. 7). In addition to the core P-element, the surrounding AA composition is of relevance, potentially indicating that this area acquires a structure. This could occur already in the cytosol, or as it has been speculated previously, upon contact with the chloroplast envelope^41^. It has been proposed that initial contact of TPs with the chloroplast envelope is mediated by the C-terminal part of the TP^42^. The C-terminus could then be integrated into the lipid-bilayer and diffuse along the chloroplast surface until the TP gets in contact with the TOC machinery^41^ and the actual import process is initiated. Since our identified P-elements are located at the C-terminus we also consider this possibility as a means of discriminating between the two chloroplast types (option 2 in Fig. 8A). Of course this would require the envelopes of the two chloroplast types to be different, for example either in their lipid composition, or by the occurrence of additional receptors. This would then generate a contact surface that is either permissive for docking or in the case of P-specific precursors trying to dock to a C-chloroplast, prevent contact.

Lastly, it is possible that discrimination of the P-specific element does not occur prior to entry of the precursor into the chloroplasts. Instead, a chloroplast type specific protease that detects and subsequently degrades all precursors which are tagged with a P-specific element could be located specifically in the C-chloroplasts (option 3 in Fig. 8A). We can also not exclude that cytosolic or chloroplastic chaperones could be involved in transport, recognition and degradation of all three options discussed.

We consistently observed correct targeting for P- but not for C-localized proteins which could indicate different targeting mechanisms for the two protein classes. It has been demonstrated that the unique SCC4 related subcellular compartmentalization and the morphological and biochemical specialization of the two chloroplast types develop gradually from very young towards more mature cells^13^,^14^,^15^,^43^ (Fig. 8B). For example, very young cells from the base of young leaves do not yet show a clear separation into P- and C-chloroplasts. Instead, chloroplasts appear monomorphic and mRNA *in situ* hybridization as well as immunolocalization studies indicate that all chloroplasts at this stage express and accumulate Rubisco large and small subunit, whereas at this stage there are very low levels of PPDK and other C4 cycle related proteins such as PEPC. In contrast, more developed cells from the midsection of young leaves already show the positioning of P- and C-chloroplasts although they still all contain Rubisco while at this stage leaves have low levels of PPDK^14^. Online isotope discrimination analysis furthermore showed that these young cells operate in a “default” C3 mode which is compatible with the lack of biochemical specialization observed in this stage^14^. Taken together this data indicate that subcellular positioning of P- and C-chloroplasts occurs during development before the actual biochemical specialization. This is also compatible with the expression patterns of C3 and C4 related proteins. Whereas expression and accumulation of C3 related proteins occurs early during development, previous *in situ* localization^14^, expression^43^ and proteome data^16^ all indicate that C4 related transcripts and proteins accumulate much later during development (Fig. 8C). Accordingly, in the early stages, the sorting mechanism that keeps Rubisco out of the P-chloroplast is not yet developed. Once the subcellular development of chloroplasts in the C and P domains occurs, Rubisco is localized in both chloroplast types. Thus, it needs either to be degraded specifically in the P-chloroplasts at this stage or alternatively, import of new proteins needs to be prevented and the “normal” protein turnover (half-life time of Rubisco from other species has been estimated between a few days and a week^44^,^45^) would then remove Rubisco from the P-chloroplasts over time. Development of the blockage of entry of P-selective peptides by the central chloroplasts might occur before or immediately after initiation of the development of the two compartments. Finally at the tip of young leaves, complete partitioning of C3 and C4 related proteins in the P- and C-chloroplasts is achieved. In summary, the different timings in expression of C3 and C4 related proteins as well as the different accumulation characteristics observed previously and in this study suggest different mechanism may be responsible for targeting of P- and C-localized proteins.

It is tempting to speculate that correct C-specific RSSU targeting in young protoplasts could be achieved if levels and timing of expression would resemble the natural situation more closely. For example, it has been shown that correct targeting of different phosphate transporters in *Medicago truncatula* depends critically on the correct timing of expression which is achieved only under the control of their endogenous promoters but not under control of related promoters^46^. Accordingly, testing of *rbcS* fusions constructs under the control of their endogenous promoters in stably transformed plants would be needed to see if the sorting mechanism is also subject to temporal changes in the SCC4 system. However, neither stable transformation nor information on promoter sequences are currently available for Bienertia.

Numerous previous studies have reported the specific localization of PPDK and RSSU in the P- and C-chloroplasts, respectively^8^,^17^,^47^. In contrast, we observed in our experiments correct targeting (for the P-proteins) only when tested in protoplasts prepared from young but not from mature leaves. This is similar to a previous study, where also specific targeting was not observed for either P- or C-targeted GFP fusions in mature Bienertia protoplasts (young protoplasts were not tested)^48^. This discrepancy can probably be explained by the different analysis methods: Previous *in situ* localization and western/proteomics analysis captured a “snapshot” of the total protein accumulation in the chloroplasts, accumulated over the lifetime of the organelle. In contrast, *in vivo* GFP localization studies also include the dynamics of the actual import reaction which might change throughout development. For example, it has been proposed that general import capacity is reduced in mature compared to younger chloroplasts^49^ and this correlates with the overall expression levels of TOC components which are generally highest in young developing chloroplasts but reduced in mature organelles^29^,^31^,^50^. However, it was later shown that the situation is a little more complex in that different substrates are imported with different efficiencies in chloroplasts of different development stages^51^. Also in Bienertia, levels of TOC 159 and TOC132 decrease from a very high level in young developing cells to a basal level towards leaf maturation^33^. Accordingly, either TOC composition or the sorting/import capacity in mature Bienertia protoplasts might be inadequate, especially when fusion constructs are massively overexpressed under the control of the 35 S promoter. It is important to note however that transcript levels of both RSSU and PPDK are still high in mature leaves^43^. As a consequence, this would mean that either these transcripts are not efficiently translated to proteins in mature cells or that expression levels are still much lower compared to expression driven by the 35S promoter. Alternatively, it is also possible that the observed failure to correctly sort proteins is caused if mature protoplasts are more prone to stress compared to younger protoplasts. For example, mature protoplasts are much bigger and damage more easily during isolation. We observed frequently that the structural integrity in mature protoplasts was compromised, for example, the central compartment was often not located in the center but shifted towards the periphery of the cell, which might occur if the vacuole becomes damaged. In this case, the observed differences between young and mature protoplasts in the targeting behavior of P- and C-specific proteins would not truly reflect developmental effects but rather artifacts of the analysis method.

## Conclusion

SCC4 species such as Bienertia represent not only a new way to perform a carbon concentrating cycle within individual cells in terrestrial plants; they are also an exciting puzzle for cell biologists. The occurrence of the same type of organelle in two specialized forms within individual cells suggests this requires novel mechanisms of intracellular protein sorting, as well as regulators of development and positioning of organelles. While we provide in this study the first step towards understanding this phenomenon, many questions remain unsolved. For example, the historical ‘hen-egg’ problem underlies all sorting mechanisms discussed here to some extent: If specific transporters, proteases or other unknown mechanism facilitate the selective accumulation in one or the other chloroplast types, how do these “determinants” reach their correct localization in the first place? Especially in plants, little is known about subcellular organization in the developmental context. The successful establishment of stable transformation technology together with detailed information on the genome of SCC4 species will be crucial to address this and other questions in the future.

## Methods

### Plant growth conditions

Seeds of *B. sinuspersici* were planted in soil and grown under controlled conditions in a GroBank Chamber (CLF Plant Climatics, Germany) at a day/night temperature of 25/20 °C. After germination for one day in the dark, seedlings were illuminated with a 16/8 h light/dark cycle at a photon flux intensity of 250 μmol m^−2^ s^−1^. Plants were shifted after two weeks into a growth cabinet with a day/night temperature of 30/18 °C with 60% relative humidity and a photoperiod of 16/8 h light/dark at a photon flux intensity of 350 μmol m^−2^ s^−1^. Plants were watered twice a week with 0.03 M NaCl and 0.001% (v/v) Wuxal fertilizer (Manna, Germany) and used after growing for 3 months for protoplast isolation.

### Plasmid construction

Subcellular localization was visualized by GFP-fusion proteins. GFP-fusion constructs were generated utilizing the 35 S:puc18-spGFP6 expression vector^48^. Different sequences were fused at the 5′ end of GFP using *Xma*I/*Spe*I restriction sites (Table S1). For all experiments with endogenous Bienertia sequences, DNA fragments were amplified by PCR from cDNA, generated from Bienertia mRNA isolated by the GeneJET Plant RNA Purification Kit (Thermo Fisher Scientific, USA). Oligonucleotides (Metabion, Germany) used for amplification contained additional *Xma*I/*Spe*I restriction sites (Table S2). Vector and PCR fragments were digested with *Xma*I/*Spe*I and ligated into the backbone with T4 DNA Ligase (Thermo Fisher Scientific, USA). All constructs were verified by sequencing (Seqlab, Germany).

### Mutagenesis

Alanine substitution mutants (Table S3) were generated by ‘splicing by overlapping extension PCR’ (SOE-PCR) as described^52^ or with the QuickChange Lightning Site-Directed Mutagenesis Kit (Agilent Technologies, USA). Oligonucleotide sequences for mutagenesis are given in Table S2. For both mutagenesis techniques, the non mutated GFP-fusion constructs (TP_TPI, TP_PPDK) served as template. Complex mutagenesis constructs were generated by gene fragment synthesis service (Eurofins Genomic GmbH, Germany). Sequences are given in Table S4. All fragments contained a *Xma*I and *Spe*I restriction site on the 5′ or 3′ end for cloning into the expression vector 35 S:puc18-spGFP6^48^.

### Protoplast isolation and transient expression in protoplasts

Protoplasts of leaves from different developmental stages were utilized for localization experiments. Mature protoplasts were isolated from mature leaves (between 1.0–1.5 cm long) following an earlier protocol^17^ with slight modifications: For isolation of chlorenchyma cells, the epidermis of several mature leaves was removed by gently squeezing and rolling with a round bottom tube. Leaves were then transferred into digest buffer (1.6% (w/v) Cellulase Onozuka R-10 (Duchefa, Netherlands), 0.25% (w/v) Macerozyme R-10 (Duchefa, Netherlands), 5 mM MES-NaOH, pH 5.7, 10 mM CaCl_2_ and incubated for 1 h at 35 °C in a water bath. All buffers for protoplast isolation were supplemented with glycine-betaine in a concentration matching the internal osmolite concentration of Bienertia chlorenchyma cells as determined by a vapor pressure osmometer (Vapro 5520, Germany) to account for osmotic differences between buffer and isolated cells/protoplasts. Undigested remains of the leaves were removed with tweezers and protoplasts were centrifuged for 1 min at 51 xg in a swing-out rotor. Supernatant was discarded and the pellet was resuspended in 20% (w/v) sucrose + glycine-betaine. Protoplasts were centrifuged for 5 min at 300 xg. Intact protoplasts floating on top of the sucrose cushion were transferred to a round bottom tube. Protoplasts were washed with glycine-betaine buffer (5 mM MES-NaOH, pH 5.7, 10 mM CaCl_2_, glycine-betaine) and centrifuged for 1 min at 51 xg in a swing-out rotor. The supernatant was discarded and protoplasts were resuspended in glycine-betaine buffer.

For the isolation of young protoplasts, 0.3–0.5 cm long leaves served as source material. Leaves were cut once in longitudinal direction and transferred to a 35 mm petri dish with digestion buffer. Leaves were slowly shaken for 3 h and afterwards filtered through a 70 micron nylon mesh. Protoplasts were centrifuged for 5 min at 300 xg in a swing-out rotor and the supernatant was discarded. Protoplasts were washed with glycine-betaine buffer followed by centrifugation for 5 min at 300 xg in a swing-out rotor. The supernatant was discarded and protoplasts were resuspended in glycine-betaine buffer. Transfection of mature as well as young protoplasts was performed as described in ref. 47 but instead with 25 μg plasmid. *Arabidopsis thaliana* protoplast isolation and transfection was done as described in ref. 53.

### Confocal microscopy

Confocal microscopy was performed using a Nikon Eclipse TE2000-E laser scanning confocal microscope (Nikon, Germany). Images were acquired through a Nikon Plan Apo, 60x/1,20 objective at a maximum digital resolution of 1024 × 1024 pixels. The fluorescence of GFP was analyzed by excitation at 488 nm and emission was detected between 509/26 nm. Chlorophyll autofluorescence was analyzed by excitation at 408 nm and emission was detected between 620/700 nm. Image processing was performed using Fiji^54^.

### Fluorescence microscopy

Fluorescence microscopy was performed using a Nikon Eclipse Ti fluorescence microscope (Nikon, Germany). Images were acquired through a Nikon Plan Apo, 40x/0.95 objective. The fluorescence of GFP was analyzed by excitation at 480/20 nm and emission was detected between 510/20 nm. Chlorophyll autofluorescence was analyzed by excitation of 550/75 nm and emission was detected between 590/675 nm. In order to quantify as many transformed protoplasts as possible, large composite pictures (0.4 mm × 0.4 mm at 400× magnification) of droplets containing protoplasts were acquired automatically (with an overlap of 2% between each individual picture) and saved for later quantification (see next section). Image processing was performed using NIS-Elements AR 4.40.00 (Nikon, Germany).

### Quantification

For quantitative analysis, transformed protoplasts identified from automatically generated composite pictures were categorized into “P-specific”, “general import” or “cytosolic”. At least three completely independent biological replicates, conducted on different days with different batches of protoplasts for each construct tested, were analyzed. Differences for quantitative data were additionally tested statistically for significance to reduce noise from variability in the expression patterns of individual protoplasts. Therefore, count data was expressed relative to WT_TPI and WT_PPDK, respectively and ratios were then transformed to arcsine to achieve normal distribution, followed by a two-tailed Student’s t-test. Generally, all situations to be compared were performed “blind” in order to avoid human bias from interpreting the microscopic results. Therefore, the experimenter performing the microscopic classifications was unaware of the nature of the construct he/she was observing. Only after quantification, the identity of the sample was revealed.

## Additional Information

**How to cite this article**: Wimmer, D. *et al*. Transit peptide elements mediate selective protein targeting to two different types of chloroplasts in the single-cell C4 species *Bienertia sinuspersici. Sci. Rep.* **7**, 41187; doi: 10.1038/srep41187 (2017).

**Publisher's note:** Springer Nature remains neutral with regard to jurisdictional claims in published maps and institutional affiliations.

## Supplementary Material

Supplemental Material
