# Supplementary material for: Transit peptide elements mediate selective protein targeting to two different types of chloroplasts in the single-cell C4 species Bienertia sinuspersici
Source: Sci Rep. 2017 Jan 23;7:41187. doi: 10.1038/srep41187 (PMC5253730; doi:10.1038/srep41187)
Supplement: Supplemental Material [file srep41187-s1.pdf]

Transit peptide elements mediate selective protein targeting to two different types of chloroplasts in the single-cell C4 species *Bienertia sinuspersici*

Diana Wimmer, Philipp Bohnhorst, Vinay Shekhar, Inhwan Hwang and Sascha Offermann

Supplemental Material

Tab. S1. DNA sequences used for localization studies with GFP-fusion constructs

All gene fragments used for localization studies were cloned to the 5' end of GFP. ppdk – pyruvate; Pi-dikinase; rbcS – Rubisco small subunit; tpi – triosephosphate isomerase; ak – adenylate kinase, TP_ppdk – transit peptide of pyruvate; Pi-dikinase; TP_tpi – transit peptide of triosephosphate isomerase; TP_ak – transit peptide of adenylate kinase

ppdk224aa

gctacccggg**ATG**GCATTATGTTTCAAAGGAATGCTAATCAGATCTGCTCCAGATGTATTTACACATACACTTGGTTATATGAAGGACCAATATCAAGTTGGTTGTAGCCAATGCAATAGTTTTCAGCGTGTTCAATTCCGGAACAGGAGGAGATGCCCACATCGATTAACTAGCCAGAGCCAGTCCAACAGACAA
GATGTCATGGCTTTGATCTCAGACCCAGTTTCAACCACCACACAGCAGCGAGTATTTACCTTTGGTAAAGGAAGAAGTGACGGGGACAAGAGCATGAAGTCCTTGTTGGGAGGTAAAGGAGCAAATCTTGCAGAAATGGCAAGCATAGGTTTATCTGTTCCTCCTGGGTTGACCATTTCTACAGAAGCATGCCAAG
AGTATCAGGACAGTGGCAAAATGCTTCCTGAAAGTCTGTGGGAGGAAATCCTTGAGGGCTTGAGGGTTATAGAGAGTGACATGGGAGCATACCTTGGAGACTCCTCTACACCTCTTCTACTTTCAGTTCGTTCTGGTGCTGCGATTTCTATGCCAGGGATGATGGACACTATCCTTAATCTTGGACTTAATGATGA
AGTAGTTTCAGGGCTCGCCGCAAAGAGTGGAGAACGCTTTGCCTATGATTCATTTAGGCGCTTCTTGGACATGTTTGGTTGTGTAGTCATGGGTactagttgc

rbcS

atacccggg**ATG**GCTTCCAGTTTGATGTCCAGCGCTGCCACTGCTGCCGTTGCTACCGCCGCTAGTGCTCAGGCAAGCATGGTGGCATCATTCAACGGGTTGAAGTCCACTTCAGCTTTCCCTGTTACCAGGAAATCCGACAATGACATTACTTCTCTTCCTAGCAACGGTGGAAAAGTCCAATGCATGCAGGTGTG
GCCACCAATTGGCAAGAAGAAGTTCGAGACTCTTTCTTACCTTCCACCTCTATCTACAGAATCCTTGTTGCGTGAGATCCAATACCTTCTTAACAAAGGTTGGGTACCCTGCTTAGAATTCGAGCCAGAACACGGATTTGTGTACCGTGAGAACCACAGGTCACCAGGGTACTATGATGGACGCTACTGGACCATGT
GGAAGCTCCCCATGTTCGGATGCACTGACCCAGCTCAGGTTGTGAATGAGCTCGAGGAGGCCAAGAAGGCTTACCCACAAGCCTTCATCCGGATCATTGGGTTCGACAACGTGCGTCAAGTCCAGTGCATCAGTTTCATTGCCTACAAGCCCCCAGGCTACataactagt

tpi

atacccggg**ATG**GCGGTTCTCTCAACTTCATTAGCGTCGCGGTTGACTAACCCTAACTCTGTTGTTTCTACTCAGTTTTCTGGTCTACGACCGTCGGTTCTTAAGTTTGAAAGTAATTCTGTTTCGAATCAGAGCTTTTTCCCGAATGTTGATTCTCAATTGCGCTTATCATCTTCTCGCCGTGGTTCTAGAGGTGT
TGTACCTATGGCTGCTTCTGGAAAGTTTTCTGGTATTCGACCATCGTTTCATAAGTTTGAAAGTAGTTCTGTTTCGAATCAAAGCTTTTTCCAGAATGTCGACTCTCATTTGCGCTTATCATCTTCTCGCCGTGGTTCTAGAGATGTTGCAGCTATGGCTGCTTCTGGAAAGTTCTTCGTTGGCGGTAATTGGAAGT
GTAATGGGACCAAAGAATCAATCACTAAGCTTGTTTCAGACTTGAACAGTGCAACATTGGAGGCTGATGTTGATGTTGTTGTCGCACCTCCGTTTGTTTACATTGATCAAGTTAAGAATTCTCTGACCAGTCGAGTAGAGATGTCAGCACAAAACTGTTGGATTGGGAAAGGTGGGGCTTTTACGGGAGAAATCTGT
GCTGAACAATTGAAGGATCTTGGGTGCCAGTGGGTTATTCTTGGGCATTCTGAGCGAAGACATGTTATTGGGGAAAATGATGAGTTTATAGGGAAAAAGGCTGCATATGCTTTGAACCAAGGTCTTGGAGTAATAGCATGTATTGGAGAGCTGTTAGAAGAAAGAGAAGCAGGAAAAACCTTTGATGTATGCTACCA
ACAATTGAAGGCTTTTGCAGATGCCGTTCCTAGCTGGGATAAGGTTGTCATTGCTTATGAACCTGTATGGGCTATTGGTACTGGCAAAGTAGCTACCCCAGAGCAGGCTCAAGAAGTGCATGTTGCTGTTCGGAATTGGCTTAAAAAGAATGTTTCTGAAGAAGTTGCTTCTAAGATGCGCATCATTTATGGAGGGT
CTGTTAATGGAGGCAACTGTGCAGAGCTTGCGAAACAAGAAGATATAGATGGATTTCTTGTGGGAGGTGCTTCTTTGAAGGGCCCTGAGTTCGCCACCATTGCTAATTCTGTGACAGCAAAGAAAGTTGCTGCCactagttat

Ak

aacccggg**ATG**AATTCCTCTACCATTTATGGAAAGCCTAATTCCGCCATTCTCTCACCCCCTTCTCCTTTTTCCCGCACTTCCTCTCCTAAATTCTCTTCAGTCTCCTTCAATTCTCATCCCTCCCTCTCCTTTCGCTTCGATCAATTAGCAACTTCACATAAGTCACGCATTCATCATCGTCATCGTCATCGTCAT
CGTCGTTCCAATTTTGTGGCTATGGCATCTACTGATAACTCAAACCCACTGGGGATTATGATATCGGGTGCTCCAGCATCTGGCAAAGGGACTCAATGTGAGCTCATAACTGAGAAATATGGTTTGGTGCATATTGCTGCTGGAGATTTGCTTCGGACAGAGATCAAATCTGGTAGTGAAAATGGAAAACAAGCCAA
GGAATACATGGACAAAGGACAGTTGGTTCCAGATGAAATAGTTGTAAAGATGGTTAAAGACCGTTTGCTGCAATCAGATTCACAAGAGAACGGCTGGCTTTTGGATGGATACCCAAGAAGCTTATCTCAAGCAATTGCTCTCAAGGACTTTGGGCTCCAACCTGACCTTTTCATTCTTTTGGAAGTCCCTGAAGAAT
TACTTGTTGAGAGGGTTGTTGGACGAAGGTTAGATCCTGATACTGGAAAAATATACCACATGAAGTATTCCCCTCCTGAGAATGATGAAGTTGCTTCCAGGCTCACTCAGCGGTTTGATGATACCGAGGAAAAGGTAAAATTGCGTCTGCAAACTTACCATCAAAATGTGGAGGCAGTGCTTTCCATGTATGAAGAC
ATAATTTTTAAGGTTGATGGAAGTGTTAAAAAAGATGATGTATTTGCTCAGATCGACAAGGCACTCTCCAAGCTTGTGGAACAAAAAACTGCTTCAAAATCGGCGGCAGCCactagttt

TP_ppdk

gctacccggg**ATG**GCATTATGTTTCAAAGGAATGCTAATCAGATCTGCTCCAGATGTATTTACACATACACTTGGTTATATGAAGGACCAATATCAAGTTGGTTGTAGCCAATGCAATAGTTTTCAGCGTGTTCAATTCCGGAACAGGAGGAGATGCCCACATCGATTAACTAGCCAGAGCCAGTCCAACAGACAAG
ATGTCATGGCTTTGATCTCAGACCCAGTTTCAActagttcg

TP_tpi

gctacccggg**ATG**GCATTATGTTTCAAAGGAATGCTAATCAGATCTGCTCCAGATGTATTTACACATACACTTGGTTATATGAAGGACCAATATCAAGTTGGTTGTAGCCAATGCAATAGTTTTCAGCGTGTTCAATTCCGGAACAGGAGGAGATGCCCACATCGATTAACTAGCCAGAGCCAGTCCAACAGACAAG
ATGTCATGGCTTTGATCTCAGACCCAGTTTCAActagttcg

TP_ak

aacccggg**ATG**AATTCCTCTACCATTTATGGAAAGCCTAATTCCGCCATTCTCTCACCCCCTTCTCCTTTTTCCCGCACTTCCTCTCCTAAATTCTCTTCAGTCTCCTTCAATTCTCATCCCTCCCTCTCCTTTCGCTTCGATCAATTAGCAACTTCACATAAGTCACGCATTCATCATCGTCATCGTCATCGTCAT
CGTCGTTCCAATTTTGTGGCTATGaaactagt

Tab. S2. Oligonucleotide sequences

Oligonucleotides for the amplification of endogenous genes and mutagenesis via SOE-PCR, QuickChange mutagenesis and PCR based mutagenesis.

SOE-PCR

Oligo name	Sequence (5' to 3')
TPI_II_fw	TCAACTTCATTAGCGGCGGCGGCGGCTGCCGCTGCCGCTGTTGTTTCTACT
TPI_II_rv	AGTAGAAACAACAGCGGCAGCGGCAGCCGCCGCCGCCGCTAATGAAGTTGA
TPI_III_fw	AACTCTGTTGTTGCTGCTGCGGCTGCTGCTGCAGCACCGTCGGTTCTT
TPI_III_rv	AAGAACCGACGGTGCTGCAGCAGCAGCCGCAGCAGCAACAACAGAGTT
TPI_IV_fw	CAGTTTTCTGGTGCAGCAGCGGCGGCTGCTGCGGCTGAAAGTAATTCT
TPI_IV_rv	AGAATTACTTTCAGCCGCAGCAGCCGCCGCTGCTGCACCAGAAAACTG
TPI_V_fw	GTTCTTAAGTTTGCAGCTGCTGCTGCTGCGGCTGCGAGCTTTTTCCCG
TPI_V_rv	CGGGAAAAAGCTCGCAGCCGCAGCAGCAGCAGCTGCAAACTTAAGAAC
TPI_VI_fw	GTTTCGAATCAGGCCGCTGCCGCGGCTGCTGCTGCTCAATTGCGCTTA
TPI_VI_rv	TAAGCGCAATTGAGCAGCAGCAGCCGCGGCAGCGGCCTGATTCGAAAC
TPI_VII_fw	AATGTTGATTCTGCAGCGGCCGCAGCAGCTGCTGCCCGTGGTTCTAGA
TPI_VII_rv	TCTAGAACCACGGGCAGCAGCTGCTGCGGCCGCTGCAGAATCAACATT
TPI_QLRL_fw	AATGTTGATTCTGCAGCAGCAGCATCATCTTCTCGC
TPI_QLRL_rv	GCGAGAAGATGATGCTGCTGCTGCAGAATCAACATT
TPI_SSS_fw	GATTCTCAATTGCGCTTAGCAGCTGCTCGCCGTGGTTCTAGAGG
TPI_SSS_rv	CCTCTAGAACCACGGCGAGCAGCTGCTAAGCGCAATTGAGAATC
PPDK_II_fw	TTCAAAGGAATGGCTGCCGCTGCTGCTGCTGCTGCATTTACACATACA
PPDK_II_rv	TGTATGTGTAAATGCAGCAGCAGCAGCAGCGGCAGCCATTCCTTTGAA
PPDK_III_fw	GCTCCAGATGTAGCAGCTGCTGCAGCTGCTGCTGCGAAGGACCAATAT
PPDK_III_rv	ATATTGGTCCTTCGCAGCAGCAGCTGCAGCAGCAGCTACATCTGGAGC
PPDK_IV_fw	ACACTTGGTTATATGGCTGCTGCTGCTGCAGCTGCTGCTAGCCAATGCAAT
PPDK_IV_rv	ATTGCATTGGCTAGCAGCAGCTGCAGCAGCAGCAGCCATATAACCAAGTGT
PPDK_V_fw	CAAGTTGGTTGTGCCGCTGCTGCTGCTGCTGCGGCTGTTCAATTCCGG
PPDK_V_rv	CCGGAATTGAACAGCCGCAGCAGCAGCAGCAGCGGCACAACCAACTTG
PPDK_VI_fw	AGTTTTCAGCGTGCTGCAGCAGCAGCCGCAGCAGCATGCCCACATCGA
PPDK_VI_rv	TCGATGTGGGCATGCTGCTGCGGCTGCTGCTGCAGCACGCTGAAAACT
PPDK_VII_fw	AACAGGAGGAGAGCCGCAGCTGCAGCAGCTGCCGCGAGCCAGTCCAAC
PPDK_VII_rv	GTTGGACTGGCTCGCGGCAGCTGCTGCAGCTGCGGCTCTCCTCCTGTT
PPDK_VIII_fw	TTAACTAGCCAGGCAGCAGCAGCCGCAGCAGCTGCCATGGCTTTGATC
PPDK_VIII_rv	GATCAAAGCCATGGCAGCTGCTGCGGCTGCTGCTGCCTGGCTAGTTAA
PPDK_VQFR_fw	AGTTTTCAGCGTGCTGCAGCCGCGAACAGGAGGAGA
PPDK_VQFR_rv	TCTCCTCCTGTTCGCGGCTGCAGCACGCTGAAAACT
PPDK_NRRR_fw	GTTCAATTCCGGGCCGCAGCAGCATGCCCACATCGA
PPDK_NRRR_rv	TCGATGTGGGCATGCTGCTGCGGCCCGGAATTGAAC

QuickChange Mutagenesis

Oligo name	Sequence (5' to 3')
TPI_RL_fw	GATTCTCAATTGGCCGCATCATCTTCTCGCCGTGGT
TPI_RL_rv	ACCACGGCGAGAAGATGATGCGGCCAATTGAGAATC
TPI_QL_fw	AATGTTGATTCTGCAGCGCGCTTATCATCTTCTCGCCGT
TPI_QL_rv	ACGGCGAGAAGATGATAAGCGCGCTGCAGAATCAACATT
TPI_R_fw	CCCGAATGTTGATTCTCAATTGGCCTTATCATCTTCTC
TPI_R_rv	GAGAAGATGATAAGGCCAATTGAGAATCAACATTCGGG

PCR-based mutagenesis

Oligo name	Sequence (5' to 3')
TPI_I_fw	atacccgggATGGCGGCTGCCGCAGCTGCAGCAGCGTCG
TPI_VIII_rv	ataactagtAGAAGCAGCCATAGCTGCAGCAGCTGCAGCAGCAGCGCG
PPDK_I_fw	atacccgggATGGCAGCAGCAGCAGCAGCAGCGGCAATCAGATCTGCT
PPDK_IX_rv	aaaactagtTGAAGCTGCGGCTGCGGCCGCTGCCGCGACATCTTGTCT
TPI_RGSR_rv	ataactagtAGAAGCAGCCATAGCTGCAGCAGCTCTAGAACC
TPI_GVVP_rv	ataactagtAGAAGCAGCCATAGGTACAACACCTGCAGCAGCAGCGCGAGA

PCR endogenous sequences

Oligo name	Sequence (5' to 3')
35S_fw	ACAATCCCACTATCCTTCGCA
GFP_rv	TCACCTTCACCCTCTCCACT
TPI_fw	atacccgggATGGCGGTTCTCTCAACTTCA
TPI_rv	ataactagtGGCAGCAACTTTCTTTGCTGT
TP_TPI_rv	ataactagtAGAAGCAGCCATAGGTACAACACC
AK_fw	aacccgggATGAATTCCTCTACCATTTA
AK_rv	aaactagtGGCTGCCGCCGATT
TP_AK_rv	aaactagtCATAGCCACAAAATTGGAACGACG
PPDK_fw	gctacccgggATGGCATTATGTTTCAAAGGAATGCTAATCAG
PPDK_rv	gcaactagtACCCATGACTACACAACCAAAC
TP_PPDK_rv	cgaactagtTGAAACTGGGTCTGAGATCAAAGC
rbcS_fw	atacccgggATGGCTTCCAGTTTGATGTCC
rbcS_rv	ataactagtGTAGCCTGGGGGCTTGTAGGC
TP_rbcS_rv	ataactagtCATGCATTGGACTTTTCCACCGTTGC

Tab. S3. Sequences of mutated gene fragments generated by SOE-PCR or QuickChange mutagenesis

All mutated gene fragments used for localization studies were cloned to the 5’end of GFP. Small letters – restriction sites; ATG is marked in bold

Method SOE-PCR:

tpi_II

atacccggg**ATG**GCGGTTCTCTCAACTTCATTAGCGGCGGCGGCGGCTGCCGCTGCCGCTGTTGTTTCTACTCAGTTTTCTGGTCTACGACCGTCGGTTCTTAAGTTTGAAAGTAATTCTGTTTCGAATCAGAGCTTTTTCCCGAATGTTGATTCTCAATTGCGCTTATCATCTTCTCGCCGTGGTTCTAGAGGTG
TTGTACCTATGGCTGCTTCTactagttat

tpi_III

Atacccggg**ATG**GCGGTTCTCTCAACTTCATTAGCGTCGCGGTTGACTAACCCTAACTCTGTTGTTGCTGCTGCGGCTGCTGCTGCAGCACCGTCGGTTCTTAAGTTTGAAAGTAATTCTGTTTCGAATCAGAGCTTTTTCCCGAATGTTGATTCTCAATTGCGCTTATCATCTTCTCGCCGTGGTTCTAGAGGTG
TTGTACCTATGGCTGCTTCTactagttat

tpi_V

atacccggg**ATG**GCGGTTCTCTCAACTTCATTAGCGTCGCGGTTGACTAACCCTAACTCTGTTGTTTCTACTCAGTTTTCTGGTCTACGACCGTCGGTTCTTAAGTTTGCAGCTGCTGCTGCTGCGGCTGCGAGCTTTTTCCCGAATGTTGATTCTCAATTGCGCTTATCATCTTCTCGCCGTGGTTCTAGAGGTG
TTGTACCTATGGCTGCTTCTactagttat

tpi_VI

atacccggg**ATG**GCGGTTCTCTCAACTTCATTAGCGTCGCGGTTGACTAACCCTAACTCTGTTGTTTCTACTCAGTTTTCTGGTCTACGACCGTCGGTTCTTAAGTTTGAAAGTAATTCTGTTTCGAATCAGGCCGCTGCCGCGGCTGCTGCTGCTCAATTGCGCTTATCATCTTCTCGCCGTGGTTCTAGAGGTG
TTGTACCTATGGCTGCTTCTactagttat

tpi_VII

atacccggg**ATG**GCGGTTCTCTCAACTTCATTAGCGTCGCGGTTGACTAACCCTAACTCTGTTGTTTCTACTCAGTTTTCTGGTCTACGACCGTCGGTTCTTAAGTTTGAAAGTAATTCTGTTTCGAATCAGAGCTTTTTCCCGAATGTTGATTCTGCAGCGGCCGCAGCAGCTGCTGCCCGTGGTTCTAGAGGTG
TTGTACCTATGGCTGCTTCTactagttat

ppdk_II

gctacccggg**ATG**GCATTATGTTTCAAAGGAATGGCTGCCGCTGCTGCTGCTGCTGCATTTACACATACACTTGGTTATATGAAGGACCAATATCAAGTTGGTTGTAGCCAATGCAATAGTTTTCAGCGTGTTCAATTCCGGAACAGGAGGAGATGCCCACATCGATTAACTAGCCAGAGCCAGTCCAACAGACAA
GATGTCATGGCTTTGATCTCAGACCCAGTTTCAactagttcg

ppdk_III

gctacccggg**ATG**GCATTATGTTTCAAAGGAATGCTAATCAGATCTGCTCCAGATGTAGCTGCTGCTGCAGCTGCTGCTGCGAAGGACCAATATCAAGTTGGTTGTAGCCAATGCAATAGTTTTCAGCGTGTTCAATTCCGGAACAGGAGGAGATGCCCACATCGATTAACTAGCCAGAGCCAGTCCAACAGACAA
GATGTCATGGCTTTGATCTCAGACCCAGTTTCAactagttcg

ppdk_IV

gctacccggg**ATG**GCATTATGTTTCAAAGGAATGCTAATCAGATCTGCTCCAGATGTATTTACACATACACTTGGTTATATGGCTGCTGCTGCAGCTGCTGCTAGCCAATGCAATAGTTTTCAGCGTGTTCAATTCCGGAACAGGAGGAGATGCCCACATCGATTAACTAGCCAGAGCCAGTCCAACAGACAA
GATGTCATGGCTTTGATCTCAGACCCAGTTTCAactagttcg

ppdk_V

gctacccggg**ATG**GCATTATGTTTCAAAGGAATGCTAATCAGATCTGCTCCAGATGTATTTACACATACACTTGGTTATATGAAGGACCAATATCAAGTTGGTTGTGCCGCTGCTGCTGCTGCTGCGGCTGTTCAATTCCGGAACAGGAGGAGATGCCCACATCGATTAACTAGCCAGAGCCAGTCCAACAGACAA
GATGTCATGGCTTTGATCTCAGACCCAGTTTCAactagttcg

ppdk_VI

Gctacccggg**ATG**GCATTATGTTTCAAAGGAATGCTAATCAGATCTGCTCCAGATGTATTTACACATACACTTGGTTATATGAAGGACCAATATCAAGTTGGTTGTAGCCAATGCAATAGTTTTCAGCGTGCTGCAGCAGCAGCCGCAGCAGCATGCCCACATCGATTAACTAGCCAGAGCCAGTCCAACAGACAA
GATGTCATGGCTTTGATCTCAGACCCAGTTTCAactagttcg

ppdk_VII

Gctacccggg**ATG**GCATTATGTTTCAAAGGAATGCTAATCAGATCTGCTCCAGATGTATTTACACATACACTTGGTTATATGAAGGACCAATATCAAGTTGGTTGTAGCCAATGCAATAGTTTTCAGCGTGTTCAATTCCGGAACAGGAGGAGAGCCGCAGCTGCAGCAGCTGCCGCGAGCCAGTCCAACAGACAA
GATGTCATGGCTTTGATCTCAGACCCAGTTTCAactagttcg

Tab. S3. Sequences of mutated gene fragments generated by SOE-PCR or QuickChange mutagenesis
All mutated gene fragments used for localization studies were cloned to the 5'end of GFP. small letters – restriction sites; ATG is marked in bold

Method SOE-PCR

tpi_SSS
atacccggg**ATG**GCGGTTCTCTCAACTTCATTAGCGTCGCGGTTGACTAACCCTAACTCTGTTGTTTCTACTCAGTTTTCTGGTCTACGACCGTCGGTTCTTAAGTTTGAAAGTAATTCTGTTTCGAATCAGAGCTTTTTCCCGAATGTTGATTCTCAATTGCGCTTAGCAGCTGCTCGCCGTGGTTCTAGAGGTG
TTGTACCTATGGCTGCTTCTactagttat

tpi_I
tacccggg**ATG**GCGGCTGCCGCAGCTGCAGCGGCGTCGCGGTTGACTAACCCTAACTCTGTTGTTTCTACTCAGTTTTCTGGTCTACGACCGTCGGTTCTTAAGTTTGAAAGTAATTCTGTTTCGAATCAGAGCTTTTTCCCGAATGTTGATTCTCAATTGCGCTTATCATCTTCTCGCCGTGGTTCTAGAGGTGT
TGTACCTATGGCTGCTTCTactagttat

ppdk_I
aatacccggg**ATG**GCAGCAGCAGCAGCAGCAGCGGCAATCAGATCTGCTCCAGATGTATTTACACATACACTTGGTTATATGAAGGACCAATATCAAGTTGGTTGTAGCCAATGCAATAGTTTTCAGCGTGTTCAATTCCGGAACAGGAGGAGATGCCCACATCGATTAACTAGCCAGAGCCAGTCCAACAGACAA
GATGTCATGGCTTTGATCTCAGACCCAGTTTCAactagttcg

ppdk_IX
gctacccggg**ATG**GCATTATGTTTCAAAGGAATGCTAATCAGATCTGCTCCAGATGTATTTACACATACACTTGGTTATATGAAGGACCAATATCAAGTTGGTTGTAGCCAATGCAATAGTTTTCAGCGTGTTCAATTCCGGAACAGGAGGAGATGCCCACATCGATTAACTAGCCAGAGCCAGTCCAACAGACAA
GATGTCGCGGCAGCGGCCGCAGCCGCAGCTTCAactagtttt

tpi_RGSR
atacccggg**ATG**GCGGTTCTCTCAACTTCATTAGCGTCGCGGTTGACTAACCCTAACTCTGTTGTTTCTACTCAGTTTTCTGGTCTACGACCGTCGGTTCTTAAGTTTGAAAGTAATTCTGTTTCGAATCAGAGCTTTTTCCCGAATGTTGATTCTCAATTGCGCTTATCATCTTCTCGCCGTGGTTCTAGAGCTG
CTGCAGCTATGGCTGCTTCTactagttat

tpi_GVVP
atacccggg**ATG**GCGGTTCTCTCAACTTCATTAGCGTCGCGGTTGACTAACCCTAACTCTGTTGTTTCTACTCAGTTTTCTGGTCTACGACCGTCGGTTCTTAAGTTTGAAAGTAATTCTGTTTCGAATCAGAGCTTTTTCCCGAATGTTGATTCTCAATTGCGCTTATCATCTTCTCGCGCTGCTGCTGCAGGTG
TTGTACCTATGGCTGCTTCTactagttat

tpi_QLRL
atacccggg**ATG**GCGGTTCTCTCAACTTCATTAGCGTCGCGGTTGACTAACCCTAACTCTGTTGTTTCTACTCAGTTTTCTGGTCTACGACCGTCGGTTCTTAAGTTTGAAAGTAATTCTGTTTCGAATCAGAGCTTTTTCCCGAATGTTGATTCTGCAGCGGCCGCATCATCTTCTCGCCGTGGTTCTAGAGGTG
TTGTACCTATGGCTGCTTCTactagttat

QuickChange Mutagenesis

tpi_QL
atacccggg**ATG**GCGGTTCTCTCAACTTCATTAGCGTCGCGGTTGACTAACCCTAACTCTGTTGTTTCTACTCAGTTTTCTGGTCTACGACCGTCGGTTCTTAAGTTTGAAAGTAATTCTGTTTCGAATCAGAGCTTTTTCCCGAATGTTGATTCTGCAGCGCGCTTATCATCTTCTCGCCGTGGTTCTAGAGGTG
TTGTACCTATGGCTGCTTCTactagttat

tpi_RL
atacccggg**ATG**GCGGTTCTCTCAACTTCATTAGCGTCGCGGTTGACTAACCCTAACTCTGTTGTTTCTACTCAGTTTTCTGGTCTACGACCGTCGGTTCTTAAGTTTGAAAGTAATTCTGTTTCGAATCAGAGCTTTTTCCCGAATGTTGATTCTCAATTGGCCGCATCATCTTCTCGCCGTGGTTCTAGAGGTG
TTGTACCTATGGCTGCTTCTactagttat

tpi_R
atacccggg**ATG**GCGGTTCTCTCAACTTCATTAGCGTCGCGGTTGACTAACCCTAACTCTGTTGTTTCTACTCAGTTTTCTGGTCTACGACCGTCGGTTCTTAAGTTTGAAAGTAATTCTGTTTCGAATCAGAGCTTTTTCCCGAATGTTGATTCTCAATTGGCCGCATCATCTTCTCGCCGTGGTTCTAGAGGTG
TTGTACCTATGGCTGCTTCTactagttat

Tab. S4

Sequences of mutated gene fragments generated by gene synthesis

All mutated gene fragments used for localization studies were cloned to the 5’ end of GFP. small letters – restriction sites; ATG is marked in bold. Italic letters in WT_TP_OOF marked the sequence of the ‘out of frame transit peptide’ of TPI.

Method gene synthesis

tpi_VII_mRNA
atacccggg**ATG**GCGGTTCTCTCAACTTCATTAGCGTCGCGGTTGACTAACCCTAACTCTGTTGTTTCTACTCAGTTTTCTGGTCTACGACCGTCGGTTCTTAAGTTTGAAAGTAATTCTGTTTCGAATCAGAGCTTTTTCCCGAATGTTGATTCTCAGCTCAGGCTTAGCAGCAGCAGACGTGGTTCTAGAGGTGT
TGTACCTATGGCTGCTTCTataactagt

tpi_VIII_mRNA
atacccggg**ATG**GCGGTTCTCTCAACTTCATTAGCGTCGCGGTTGACTAACCCTAACTCTGTTGTTTCTACTCAGTTTTCTGGTCTACGACCGTCGGTTCTTAAGTTTGAAAGTAATTCTGTTTCGAATCAGAGCTTTTTCCCGAATGTTGATTCTCAATTGCGCTTATCATCTTCTCGCAGAGGCAGCCGGGGCGT
CGTCCCGATGGCTGCTTCTactagttat

ppdk_VI_mRNA
gctacccgggg**ATG**GCATTATGTTTCAAAGGAATGCTAATCAGATCTGCTCCAGATGTATTTACACATACACTTGGTTATATGAAGGACCAATATCAAGTTGGTTGTAGCCAATGCAATAGTTTTCAGCGTGTCCAGTTTAGAAATCGACGTCGCTGCCCACATCGATTAACTAGCCAGAGCCAGTCCAACAGACAA
GATGTCATGGCTTTGATCTCAGACCCAGTTTCAactagttcg

WT_tpi_OOF
atacccggg*ATACGGCGGTTCTCTCAACTTCATTAGCGTCGCGGTTGACTAACCCTAACTCTGTTGTTTCTACTCAGTTTTCTGGTCTACGACCGTCGGTTCTTAAGTTTGAAAGTAATTCTGTTTCGAATCAGAGCTTTTTCCCGAATGTTGATTCTCAATTGCGCTTATCATCTTCTCGCCGTGGTTCTAGAGGT*
*GTTGTACCT***ATG**GCTGCTTCTATGGCTTCCAGTTTGATGTCCAGCGCTGCCACTGCTGCCGTTGCTACCGCCGCTAGTGCTCAGGCAAGCATGGTGGCATCATTCAACGGGTTGAAGTCCACTTCAGCTTTCCCTGTTACCAGGAAATCCGACAATGACATTACTTCTCTTCCTAGCAACGGTGGAAAAGTCCAATG
CATGCAGGTGTGGCCACCAATTGGCAAGAAGAAGTTCGAGACTCTTTCTTACCTTCCACCTCTATCTACAGAATCCTTGTTGCGTGAGATCCAATACCTTCTTAACAAAGGTTGGGTACCCTGCTTAGAATTCGAGCCAGAACACGGATTTGTGTACCGTGAGAACCACAGGTCACCAGGGTACTATGATGGACGCT
ACTGGACCATGTGGAAGCTCCCCATGTTCGGATGCACTGACCCAGCTCAGGTTGTGAATGAGCTCGAGGAGGCCAAGAAGGCTTACCCACAAGCCTTCATCCGGATCATTGGGTTCGACAACGTGCGTCAAGTCCAGTGCATCAGTTTCATTGCCTACAAGCCCCCAGGCTACcgaactagt

ppdk_V_VI
gctacccggg**ATG**GCATTATGTTTCAAAGGAATGCTAATCAGATCTGCTCCAGATGTATTTACACATACACTTGGTTATATGAAGGACCAATATCAAGTTGGTTGTAGCCAATGCGCTGCTGCTGCGGCTGCTGCAGCCCGGAACAGGAGGAGATGCCCACATCGATTAACTAGCCAGAGCCAGTCCAACAGACAAG
ATGTCATGGCTTTGATCTCAGACCCAGTTTCAactagttcg

ppdk_VQFR
gctacccggg**ATG**GCATTATGTTTCAAAGGAATGCTAATCAGATCTGCTCCAGATGTATTTACACATACACTTGGTTATATGAAGGACCAATATCAAGTTGGTTGTAGCCAATGCAATAGTTTTCAGCGTGCTGCAGCCGCGAACAGGAGGAGATGCCCACATCGATTAACTAGCCAGAGCCAGTCCAACAGACAAG
ATGTCATGGCTTTGATCTCAGACCCAGTTTCAactagttcg

ppdk_NRRR
gctacccggg**ATG**GCATTATGTTTCAAAGGAATGCTAATCAGATCTGCTCCAGATGTATTTACACATACACTTGGTTATATGAAGGACCAATATCAAGTTGGTTGTAGCCAATGCAATAGTTTTCAGCGTGTTCAATTCCGGGCCGCAGCAGCATGCCCACATCGATTAACTAGCCAGAGCCAGTCCAACAGACAAG
ATGTCATGGCTTTGATCTCAGACCCAGTTTCAactagttcg

ShTPI
aaacccggg**ATG**GCGGTTGTATCAACTTCATTAGCGTCGCAACTAAGTAACCCTAACGCCATTGTTTCAACTCAGTTTTCTGGTCTTCGACGATCGTTTCTTAAGCTTGAAAGTAGTTCTTCTTCTTCTTCTTCAAATCAATGCTTTTTTCAAGTTGTTGATTCTCATTTGCTCTTATCGTCTTCTCGCCGTGGTTC
TAGAGGTGTTGTTACTATGGCTGGTTCTactagttaa

ShTPI_1
aaacccggg**ATG**GCGGTTGTATCAACTTCATTAGCGTCGCAACTAAGTAACCCTAACGCCATTGTTTCAACTCAGTTTTCTGGTCTTCGACGATCGTTTCTTAAGCTTGAAAGTAGTTCTTCTTCTTCTTCTTCAAATCAATGCTTTTTTCAAGTTGTTGATTCTCAATTGCGCTTATCGTCTTCTCGCCGTGGTTC
TAGAGGTGTTGTTACTATGGCTGGTTCTactagttaa

ShTPI_2
aaacccggg**ATG**GCGGTTGTATCAACTTCATTAGCGTCGCAACTAAGTAACCCTAACGCCATTGTTTCAACTCAGTTTTCTGGTCTTCGACGATCGTTTCTTAAGCTTGAAAGTAGTTCTTCTTCTTCTTCTTCAAATCAATGCTTTTTTCAAGTTGTTGATTCTCAATTGCGCTTATCGTCTTCTCGCCGTGGTTC
TAGAGGTGTTGTTCCTATGGCTGCTTCTactagttaa

ShTPI_3
aaacccggg**ATG**GCGGTTGTATCAACTTCATTAGCGTCGCAACTAAGTAACCCTAACGCCATTGTTTCAACTCAGTTTTCTGGTCTTCGACGATCGTTTCTTAAGCTTGAAAGTAGTTCTTCTTCTTCTTCTTCAAATCAAAGCTTTTTTCCGAATGTTGATTCTCAATTGCGCTTATCGTCTTCTCGCCGTGGTTC
TAGAGGTGTTGTTCCTATGGCTGCTTCTactagttaa

ShTPI_4
aaacccggg**ATG**GCGGTTGTATCAACTTCATTAGCGTCGCAACTAAGTAACCCTAACGCCATTGTTTCAACTCAGTTTTCTGGTCTTCGACGATCGTTTCTTAAGCTTGAAAGTAATTCTGTTTCTAATCAAAGCTTTTTTCCGAATGTTGATTCTCAATTGCGCTTATCGTCTTCTCGCCGTGGTTCTAGAGGTGT
TGTTCCTATGGCTGCTTCTactagttaa

Tab. S4
Sequences of mutated gene fragments generated by gene synthesis
All mutated gene fragments used for localization studies were cloned to the 5’ end of GFP. small letters – restriction sites; ATG is marked in bold. Italic letters in WT_TP_OOF marked the sequence of the ‘out of frame transit peptide’ of TPI.

TP_TPI-TP-TP_rbcS
atacccgggATGGCGGTTCTCTCAACTTCATTAGCGTCGCGGTTGACTAACCCTAACTCTGTTGTTTCTACTCAGTTTTCTGGTCTACGACCGTCGGTTCTTAAGTTTGAAAGTAATTCTGTTTCGAATCAGAGCTTTTTCCCGAATGTTGATTCTCAATTGCGCTTATCATCTTCTCGCCGTGGTTCTAGAGGTG
TTGTAATGGCTTCCAGTTTGATGTCCAGCGCTGCCACTGCTGCCGTTGCTACCGCCGCTAGTGCTCAGGCAAGCATGGTGGCATCATTCAACGGGTTGAAGTCCACTTCAGCTTTCCCTGTTACCAGGAAATCCGACAATGACATTACTTCTCTTCCTAGCAACGGTGGAAAAGTCCAATGCATGactagttat

TP_rbcS-TP_TPI
atacccgggATGGCTTCCAGTTTGATGTCCAGCGCTGCCACTGCTGCCGTTGCTACCGCCGCTAGTGCTCAGGCAAGCATGGTGGCATCATTCAACGGGTTGAAGTCCACTTCAGCTTTCCCTGTTACCAGGAAATCCGACAATGACATTACTTCTCTTCCTAGCAACGGTGGAAAAGTCCAAATGGCGGTTCTCT
CAACTTCATTAGCGTCGCGGTTGACTAACCCTAACTCTGTTGTTTCTACTCAGTTTTCTGGTCTACGACCGTCGGTTCTTAAGTTTGAAAGTAATTCTGTTTCGAATCAGAGCTTTTTCCCGAATGTTGATTCTCAATTGCGCTTATCATCTTCTCGCCGTGGTTCTAGAGGTGTTGTACCTATGGCTGCTTCTac
tagttat

Tab. S5. ChloroP predicted transit peptide sequences of P-chloroplast localized proteins

Score - output score from the second step network (55), TP (transit peptide) length - predicted length of the precursor proteins PPDK – pyruvate; Pi-dikinase; TPI – triosephosphate isomerase; AK – adenylate kinase

Protein	Score	TP length
PPDK	0.519	72
TPI	0.583	64
AK	0.590	69

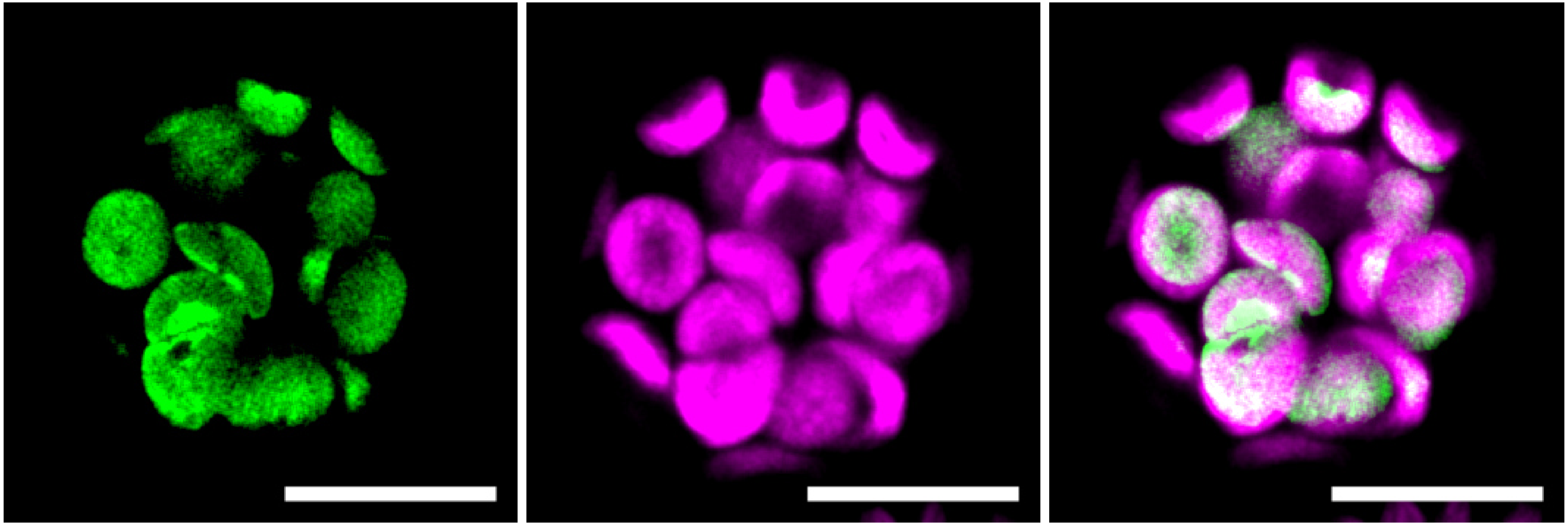

Fig. S1. Subcellular localization of PPDK in *Arabidopsis thaliana*
Confocal image of Bienertia sinuspersici PPDK224-GFP-fusion heterologously expressed in *A. thaliana* mesophyll protoplasts. The fluorescence image is shown in the GFP channel (excitation 488 nm/emission 509/525 nm) and the chlorophyll autofluorescence in the red channel (excitation 408 nm/emission 620/700 nm). Additionally, the merged channel is shown. The image is representative for n = 2 independent experiments. All scale bars = 10 μm

A

TP_TPI ::
TP_RSSU

B

TP_RSSU ::
TP_TPI

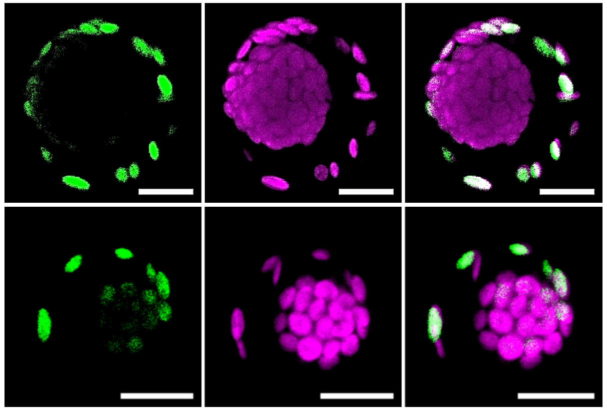

Fig. S2. Subcellular localization of fusions between the transit peptides of TPI and RSSU

A: Confocal images of *Bienertia sinuspersici* mesophyll protoplasts expressing the transit peptide of TPI fused in frame in front of the in frame transit peptide of RSSU (TP_TPI :: TP_RSSU) and **B:** the transit peptide of RSSU fused in frame in front of the transit peptide of TPI (TP_RSSU :: TP_TPI). The fluorescence image is shown in the GFP channel (excitation 488 nm/emission 509/525 nm) and the chlorophyll autofluorescence in the red channel (excitation 408 nm/emission 620/700 nm). Additionally, the merged channel is shown. All scale bars = 10 µm
